# Pneumococcal Metabolic Adaptation and Colonization Are Regulated by the Two-Component Regulatory System 08

**DOI:** 10.1128/mSphere.00165-18

**Published:** 2018-05-16

**Authors:** Alejandro Gómez-Mejia, Gustavo Gámez, Stephanie Hirschmann, Viktor Kluger, Hermann Rath, Sebastian Böhm, Franziska Voss, Niamatullah Kakar, Lothar Petruschka, Uwe Völker, Reinhold Brückner, Ulrike Mäder, Sven Hammerschmidt

**Affiliations:** a Department of Molecular Genetics and Infection Biology, Interfaculty Institute for Genetics and Functional Genomics, Center for Functional Genomics of Microbes, University of Greifswald, Greifswald, Germany; b Genetics, Regeneration and Cancer (GRC) Research Group, University Research Center (SIU), Universidad de Antioquia (UdeA), Medellin, Colombia; c Basic and Applied Microbiology (MICROBA) Research Group, School of Microbiology, Universidad de Antioquia (UdeA), Medellin, Colombia; d Department of Functional Genomics, Interfaculty Institute for Genetics and Functional Genomics, Center for Functional Genomics of Microbes, University Medicine Greifswald, Greifswald, Germany; e Department of Microbiology, University of Kaiserslautern, Kaiserslautern, Germany; University of Nebraska Medical Center

**Keywords:** Streptococcus pneumoniae, colonization, gene regulation, physiology, two-component regulatory systems

## Abstract

Streptococcus pneumoniae two-component regulatory systems (TCS) enable adaptation and ensure its maintenance in host environments. This study deciphers the impact of TCS08 on pneumococcal gene expression and its role in metabolic and pathophysiological processes. Transcriptome analysis and real-time PCR demonstrated a regulatory effect of TCS08 on genes involved mainly in environmental information processing, intermediary metabolism, and colonization by S. pneumoniae D39 and TIGR4. Striking examples are genes for fatty acid biosynthesis, genes of the arginine deiminase system, and the *psa* operon encoding the manganese ABC transport system. *In silico* analysis confirmed that TCS08 is homologous to Staphylococcus aureus SaeRS, and a SaeR-like binding motif is displayed in the promoter region of *pavB*, the upstream gene of the *tcs08* operon encoding a surface-exposed adhesin. Indeed, PavB is regulated by TCS08 as confirmed by immunoblotting and surface abundance assays. Similarly, pilus-1 of TIGR4 is regulated by TCS08. Finally, *in vivo* infections using the acute pneumonia and sepsis models showed a strain-dependent effect. Loss of function of HK08 or TCS08 attenuated D39 virulence in lung infections. The RR08 deficiency attenuated TIGR4 in pneumonia, while there was no effect on sepsis. In contrast, lack of HK08 procured a highly virulent TIGR4 phenotype in both pneumonia and sepsis infections. Taken together, these data indicate the importance of TCS08 in pneumococcal fitness to adapt to the milieu of the respiratory tract during colonization.

**IMPORTANCE** Streptococcus pneumoniae interplays with its environment by using 13 two-component regulatory systems and one orphan response regulator. These systems are involved in the sensing of environmental signals, thereby modulating pneumococcal pathophysiology. This study aimed to understand the functional role of genes subject to control by the TCS08. The identified genes play a role in transport of compounds such as sugars or amino acids. In addition, the intermediary metabolism and colonization factors are modulated by TCS08. Thus, TCS08 regulates genes involved in maintaining pneumococcal physiology, transport capacity, and adhesive factors to enable optimal colonization, which represents a prerequisite for invasive pneumococcal disease.

## Abstract (precis)

Streptococcus pneumoniae interplays with its environment by using 13 two-component regulatory systems and one orphan response regulator. These systems are involved in the sensing of environmental signals, thereby modulating pneumococcal pathophysiology. This study aimed to understand the functional role of genes subject to control by the TCS08. The identified genes play a role in transport of compounds such as sugars or amino acids. In addition, the intermediary metabolism and colonization factors are modulated by TCS08. Thus, TCS08 regulates genes involved in maintaining pneumococcal physiology, transport capacity, and adhesive factors to enable optimal colonization, which represents a prerequisite for invasive pneumococcal disease.

## INTRODUCTION

Regulatory systems are inherent features of living organisms, ensuring a rapid response and adaptation to diverse environmental conditions and acting as on/off switches for gene expression (1). Regulation in bacteria is predominantly conducted by two-component regulatory systems (TCS), quorum sensing proteins, and stand-alone regulators (2–4). TCS are the most common and widespread sensing mechanisms in prokaryotes, functioning by activation of effectors through the autophosphorylation of a conserved histidine kinase (HK) and the phosphor transfer to its cognate partner protein, also referred to as a response regulator (RR). These systems are able to sense environmental conditions and coordinate the appropriate response to ensure survival, fitness, and pathogenicity (4–9).

*In silico* and functional analysis of the pneumococcal genome identified 13 cognate HK-RR pairs and an additional orphan unpaired RR in different pneumococcal strains (10, 11). TCS in pneumococci have been associated with fitness and regulation of virulence factors, and 11 TCS are reported to contribute to pneumococcal pathogenicity (11, 12). ComDE and CiaRH, both involved in the control of competence and cell survival under stress conditions, have been studied most extensively (13–18). WalRK is another well-characterized TCS in pneumococci, featuring the only PAS (Per-Arnt-Sim) domain in Streptococcus pneumoniae and involved in maintenance of cell wall integrity by regulating the proteins PcsB and FabT (19–21). Furthermore, this system is the only TCS which has been shown to be essential for pneumococcal viability. However, it was proven later that this effect on viability was due to the regulation of the peptidoglycan hydrolase PcsB, whose loss of function leads to an unstable membrane and impaired cell viability (22, 23). Pneumococcal TCS08 (in TIGR4, genes *sp_0083* to *sp_0084* encode RR08 and HK08) is highly homologous to the SaeRS system of Staphylococcus aureus (24), where it has been associated with the regulation of genes encoding α-hemolysin (*hla*), coagulase (*coa*), fibronectin (Fn) binding proteins, and 20 other virulence factors (25–27). Interestingly, the SaeRS system of S. aureus has been shown to respond to subinhibitory concentrations of α-defensins and high concentrations of H_2_O_2_, suggesting a sensing mechanism responsive to host immune system molecules and membrane alterations (26, 27). In pneumococci, a previous study on TCS08 has revealed its importance for pneumococcal virulence (11). Moreover, two reports have shown a regulatory effect of the pneumococcal TCS08 on the *rlrA* pathogenicity islet (pilus-1 [PI-1]) and the cellobiose phosphotransfer system (PTS) (24, 28). Hence, the initial information available about this system suggests its involvement in pneumococcal adaptation, fitness, and virulence. Nevertheless, its target genes and its precise role in pneumococcal pathogenicity are yet to be defined.

## RESULTS

### Influence of TCS08 on pneumococcal growth behavior in CDM.

**FIG 1:**
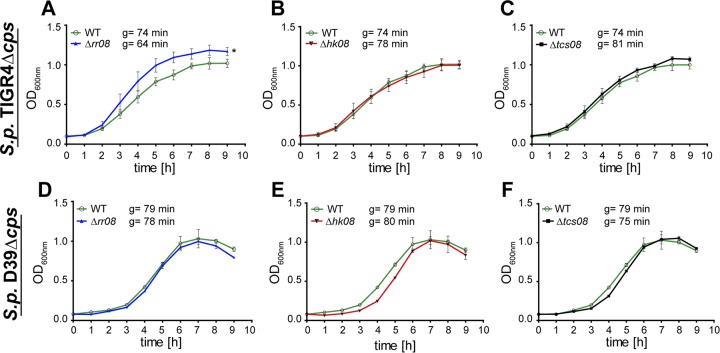
Growth behavior of pneumococcal *tcs08* mutants. Growth in CDM of S. pneumoniae TIGR4 Δ*cps* and D39 Δ*cps* parental strains versus *Δrr08* (A and D), *Δhk08* (B and E), and *Δtcs08* (C and F) mutants, respectively. The symbol “g” indicates generation time. An unpaired two-tailed *t* test was used with the generation times for statistics, and the error bars indicate the standard deviation for *n* = 3. The asterisk indicates statistical significance among the generation times of the different strains (*P* < 0.05).

To investigate the effect of loss of function of TCS08 components on pneumococcal fitness, nonencapsulated *S. pneumoniae* D39 and TIGR4 parental strains and their isogenic mutants were cultured in a chemically defined medium (CDM). All strains presented similar growth patterns and reached similar cell densities in the stationary phase, with the exception of the TIGR4 Δ*cps* Δ*rr08* mutant (Fig. 1). A steeper logarithmic phase was detected in the *rr08* mutant in TIGR4 (Fig. 1A and E). Additionally, the calculated growth rates of the different mutants in both D39 and TIGR4 strains suggested a significant reduction in the generation time of the *rr08* mutant in TIGR4 (Fig. 1A). The observed behavior among the TCS08 mutants in the CDM used in this study may point to strain-dependent specific effects.

### Impact of TCS08 on TIGR4 gene expression.

**FIG 2:**
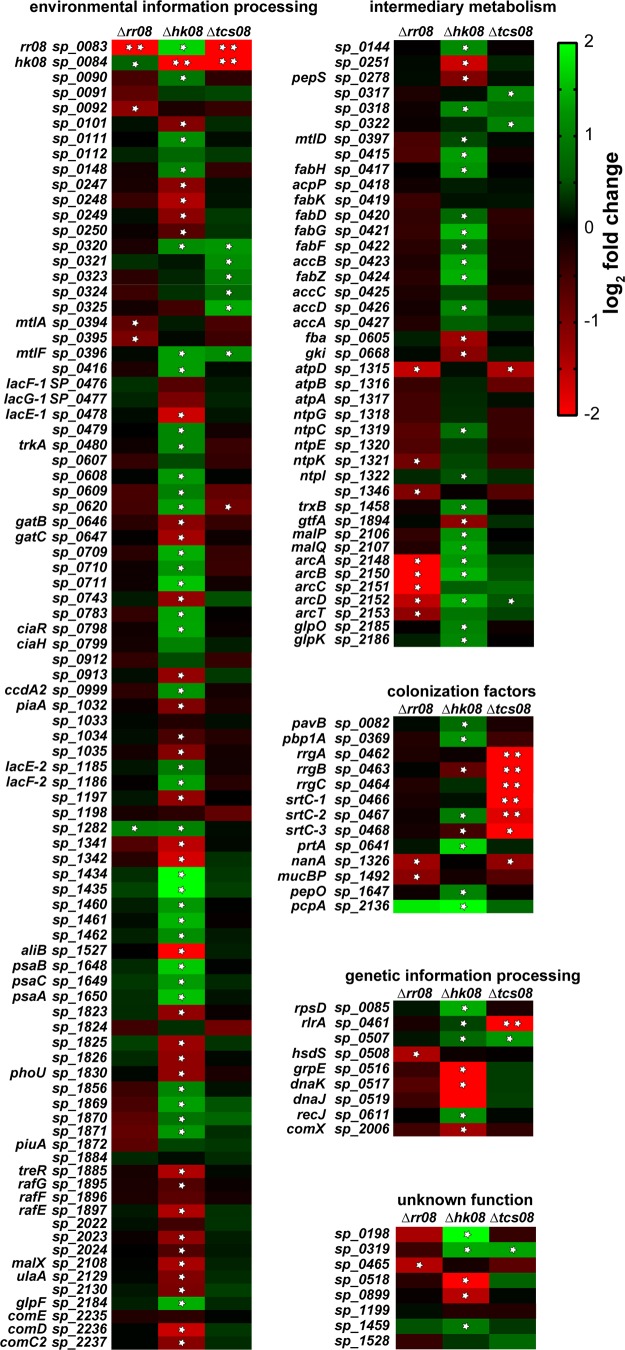
Gene expression heat map for TIGR4 wild type and isogenic *tcs08* mutants. Output of results for the microarray study using *S. pneumoniae* TIGR4 Δ*cps* and its corresponding *tcs08* mutants. The heat map indicates alterations in gene expression, where upregulation is indicated by green and downregulation is indicated by red. Single white stars indicate *P* values of <0.05, and double white stars indicate *q* values of <0.05, where *q* indicates the false discovery rate statistic result.

The initial screening for the effects of TCS08 inactivation on gene expression was conducted by microarrays using RNA samples extracted from TIGR4Δ*cps* and its isogenic *rr08*, *hk08*, and *tcs08* mutants grown in CDM. Genes presenting significant changes in gene expression higher than 2-fold with known functions or with functional domains were considered for further analysis. This led to the selection of 159 protein-encoding genes showing significant differences in expression compared to the wild type (WT) in at least one of the mutants deficient in RR08, HK08, or both (TCS08). Loss of HK08 triggered the strongest changes in expression compared to the wild type and influenced 114 genes. Differences in expression profiles of the 159 genes found in the microarray were classified by their annotated biochemical functions in 5 different categories (Fig. 2; see also Table S1 in the supplemental material). (i) The largest number of genes influenced in their expression by TCS08 was observed for genes belonging to environmental information processing (EIP). Genes belonging to this functional class are mostly involved in membrane transport by ABC transporters and phosphotransferase systems and represented 88 genes affected by mutations in TCS08. The strongest changes in gene expression within the EIP category were detected for the ABC transporters *aliB* (oligopeptide substrate-binding protein) and *sp_1434*, both in the *hk08* mutant. (ii) The second most predominant category, with 41 genes, was the intermediary metabolism (IM). Here, significant changes in the expression of genes involved in fatty acid (*fab* operon), carbon (cellobiose, mannitol, and maltose PTS), and amino acid (*arc* operon) metabolism were seen. Indeed, the absence of RR08 led to a significant reduction in the expression of the *arc* operon, involved in arginine uptake and utilization. In contrast, the expression of the *arc* genes in the strain lacking HK08 was upregulated. These changes observed in the expression of the *arc* operon were the most prominent within the IM category. (iii) Genes reported to play a role as colonization factors (CF) accounted for 13 of the 159 genes displaying expression changes in the microarray analysis. The genes found in this group encode surface-exposed proteins involved in peptidoglycan synthesis and adhesion. Among them, the gene *sp_2136*, encoding the choline-binding protein PcpA, showed the strongest upregulation in the whole-microarray analysis. The genes encoding PavB, MucBP, PepO, PrtA, and NanA displayed changes in their expression in the different *tcs08* mutants as well. These important proteins are involved in pneumococcal colonization and highlight the role of TCS08 for pneumococcal adhesion and colonization. Additionally, the lack of both components of TCS08 resulted in changes in the expression of the *rrgABC-srtC* operon, confirming the regulation of the region of diversity 4 (RD4) (identified as *rlrA* or PI-1 pathogenicity islet) by TCS08. It is noteworthy that most of these genes encode surface-displayed proteins often covalently anchored in the peptidoglycan via a transpeptidase. (iv) The fourth category encompasses genes playing a role in genetic information processing (GIP), of which 9 genes were detected as significantly influenced by TCS08. Genes like *rlrA*, *dnaK*, and *grpE* are mostly involved in DNA and protein processing. Remarkably, in the absence of both components of TCS08 a significant downregulation is seen for the positive regulator *rlrA*, involved in the expression of PI-1. (v) The last category involves genes with an unknown function (UF). Here, 8 genes out of the 159 identified genes presented changes in their expression in the microarray, including hypothetical lipoproteins like SP_0198 and SP_0899 (29). These proteins contain conserved lipobox motifs and are therefore also thought to be surface exposed and might be involved in unknown fitness-related processes.

10.1128/mSphere.00165-18.5 TABLE S1  Results of the microarray analysis. Download TABLE S1, XLS file, 0.2 MB. Copyright © 2018 Gómez-Mejia et al. 2018 Gómez-Mejia et al. This content is distributed under the terms of the Creative Commons Attribution 4.0 International license.

### TCS08 is involved in the regulation of metabolic functions of S. pneumoniae*.*

**FIG 3:**
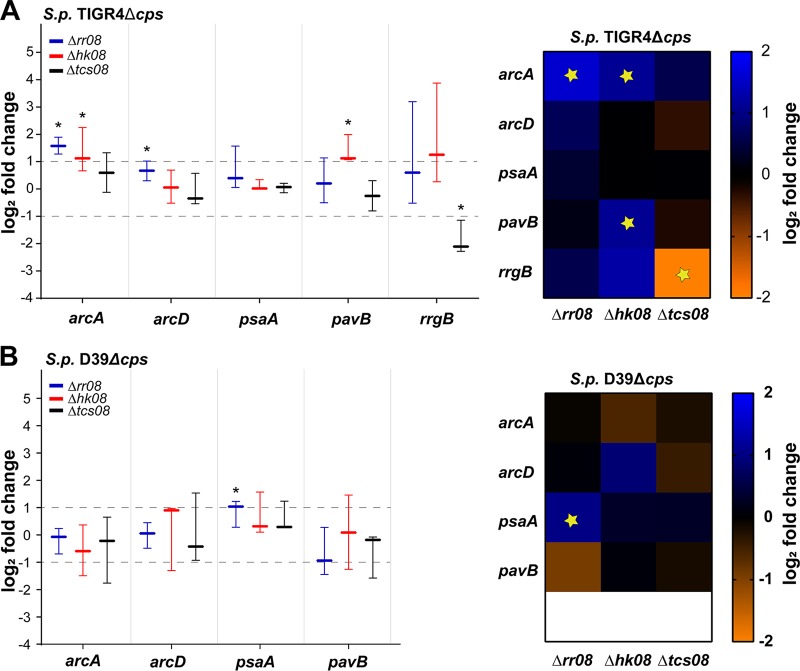
Impact of pneumococcal TCS08 on gene expression by real-time PCR. Differential gene expression in *tcs08* mutants (Δ*rr08*, Δ*hk08*, and Δ*tcs08*) analyzed by qPCR after pneumococcal cultivation in CDM. *S*. *pneumoniae* TIGR4 Δ*cps* (A) and D39 Δ*cps* (B). Specific primers for the ribosomal protein S16 (*sp_0775*) were used as normalization control. Data indicate the ΔΔ*C*_*T*_ of the fold change in the graph bar and heat map for the different *tcs08* mutants from three independent experiments. D39 Δ*cps* or TIGR4 Δ*cps* wild type was normalized to 0 and used for statistical analysis with the unpaired Student *t* test. Asterisks and yellow stars indicate *P* values of <0.05 in both the graph and the heat map for *n* = 3, respectively. Data are presented as boxes and whiskers with the median and 95% confidence intervals.

Results obtained by the microarray screening suggested a regulatory effect of TCS08 in the expression of genes involved in the uptake and transport of essential nutrients for *S. pneumoniae* TIGR4, such as arginine and manganese (Fig. 2; Table S1). These metabolites/ions are transported into the cell via specific ABC transporter systems. Of particular interest is the arginine deiminase system (ADS), which is essential for arginine uptake and utilization in pneumococci. All genes of the *arcABCDT* operon displayed important changes in their expression in the absence of the RR or HK08. Interestingly, these changes were not consistent in the two mutants, as the Δ*rr08* strain displayed a significant downregulation of this operon while the *hk08* mutant showed an upregulation (Fig. 2; Table S1). Additionally, no significant effects were observed for the *arc* operon in the Δ*tcs08* mutant. Analysis by real-time PCR (qPCR) partially confirmed the initial findings on the expression of the *arc* operon and demonstrated a strain-dependent effect for these genes. Indeed, the expression of the arginine deiminase gene *arcA* was only significantly increased in the Δ*rr08* and Δ*hk08* mutants in TIGR4 (Fig. 3A), whereas no differences were found in D39 (Fig. 3B). Furthermore, the arginine-ornithine antiporter *arcD* (30, 31) presented a similar expression as *arcA* in TIGR4 and D39 TCS08 mutants; however, the changes were not significant (Fig. 3). An additional key player in the pneumococcal fitness is the *psa* operon. This operon plays a role in the uptake of manganese and in the response to oxidative stress in the pneumococci. The analysis by microarray showed a significant increase of 2-fold in the expression of the *psa* operon for the *hk08* mutant in the TIGR4 strain (Fig. 2; Table S1). Conversely, no statistically important effects were observed in the *psa* operon in the *rr08* and *tcs08* mutants in the same strain (Fig. 2; Table S1). Validation of the microarray data by qPCR discovered a significant increase in the expression of *psaA* in the *rr08* mutant of D39. Surprisingly, the microarray data for the *psa* operon could not be confirmed by qPCR in TIGR4 (Fig. 3A).

**FIG 4:**
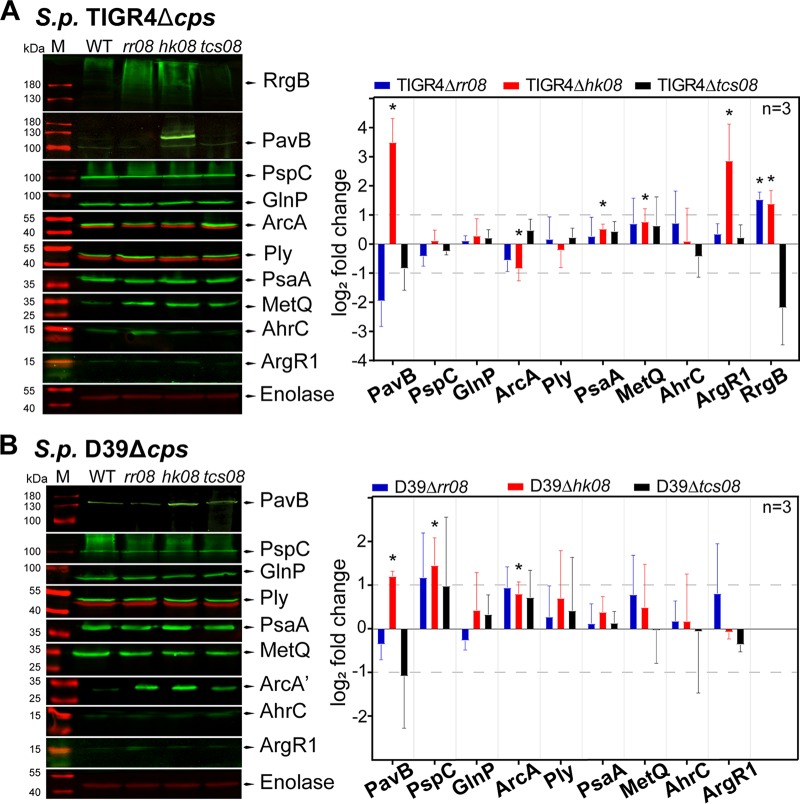
Protein expression levels in pneumococcal *tcs08*-deficient strains. Quantification of different proteins in pneumococci by immunoblotting in *S. pneumoniae* TIGR4 Δ*cps* (A) and D39 Δ*cps* (B) and their corresponding isogenic *tcs08* mutants. The unpaired Student *t* test was applied, and the enolase of D39 Δ*cps* or TIGR4 Δ*cps* was used as reference. *, *P* values < 0.05; *n* = number of biological replicates. The horizontal segmented lines indicate the 2-fold change, and the error bars indicate the standard deviation. M, molecular mass markers.

Immunoblot analyses of pneumococci cultured in CDM were carried out to elucidate the effect of TCS08 components on the protein levels of selected candidates from D39 and TIGR4 based on gene expression data (Fig. 4). For the ADS, the arginine deiminase ArcA was selected as a representative protein. Analysis of protein abundance of ArcA in D39 revealed a significant increase in the Δ*hk08* mutant (Fig. 4B). In contrast, the loss of HK08 in TIGR4 resulted in a 2-fold-lower abundance of ArcA (Fig. 4A). The remaining *rr08* and *tcs08* mutants in both strains showed nonsignificant effects in the protein levels of ArcA. Interestingly, the results obtained for the ArcA protein in the absence of HK08 in both strains did not reflect the transcriptome (2-fold upregulation) or qPCR results. In the case of PsaA, the immunoblot analysis confirmed a significantly higher expression of 1.5-fold in the TIGR4 *hk08* mutant (Fig. 4A), correlating with the microarray data (Fig. 2).

**FIG 5:**
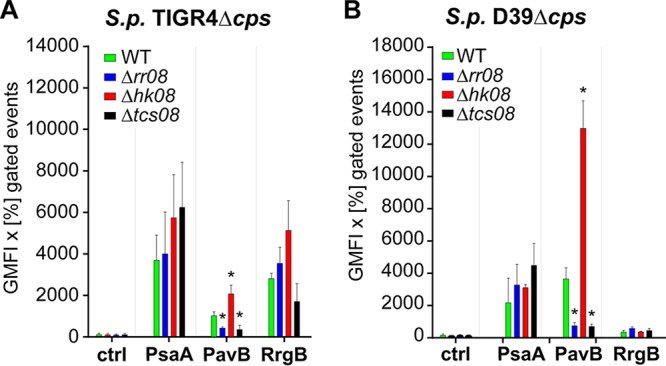
Impact of HK08 and RR08 on the abundance of pneumococcal surface proteins. The surface expression and abundance of surface proteins were analyzed by flow cytometry in *S*. *pneumoniae* TIGR4 Δ*cps* (A) and D39 Δ*cps* (B) strains and their corresponding isogenic *tcs08* mutants, all cultured in CDM. The unpaired Student *t* test was applied for the statistics, and D39 Δ*cps* or TIGR4 Δ*cps* was used as a reference accordingly. *, *P* value < 0.05 for *n* = 3. Error bars indicate the standard deviation.

In a complementary approach, the surface abundance of PsaA was examined by a flow cytometric approach (Fig. 5). For D39, a nonsignificant increase in the surface abundance of PsaA was measured in mutants lacking both TCS08 components. The modest effect of TCS08 on PsaA observed for surface abundance correlates with the immunoblot analysis (Fig. 4 and 5). Similarly, the increased surface abundance of PsaA in TIGR4 mutants lacking HK08 (Fig. 5) correlated with the immunoblot and microarray analysis.

### TCS08 regulates pneumococcal colonization factors.

The adhesins PavB and PI-1 were shown to be regulated in the TIGR4 strain by our initial microarray analysis (Fig. 2; Table S1) and confirmed by qPCR. Interestingly, *pavB* is a gene upstream of the 5′ region of the *tcs08* operon presenting properties of a sortase-anchored adhesin. PavB has been shown to interact with various extracellular matrix (ECM) proteins and probably also directly with a cellular receptor (32, 33), thereby linking pneumococci with host cells. Similarly, PI-1 is composed of the proteins RrgA, RrgB, and RrgC, with RrgB functioning as the backbone (34). The genes of *pilus-1* are part of the RD4 or *rlrA* pathogenicity island and belong to the accessory genome of some pneumococcal strains and clinical isolates, including TIGR4 (35, 36). Both PI-1 and *pavB* genes presented significant changes in gene expression with an upregulation in mutants lacking HK08 by at least 2-fold (Fig. 3). Moreover, the absence of both components of TCS08 leads to a significantly reduced expression of *pilus-1* in TIGR4, while no significant effect was seen for *pavB* in either the *rr08* or *tcs08* mutant in either the D39 or TIGR4 strain at the gene expression level.

On the protein level, quantifications were performed by immunoblotting (Fig. 4) and the levels of surface abundance were evaluated by flow cytometry (Fig. 5). For PI-1, the backbone protein RrgB was used as a representative. Immunoblot analysis and flow cytometry indicated higher protein levels and surface abundance, respectively, in mutants lacking HK08 and RR08. These results are in line with gene expression analyses. Importantly, the lower protein levels of RrgB in the absence of both TCS08 components correlated with the downregulation measured by qPCR and transcriptomics (Fig. 4A and 5A). For PavB, immunoblot assays revealed a high impact on PavB amounts in the different mutants with a 2-fold increase in the absence of HK08 in D39 and even 10-fold in TIGR4. In contrast, the lack of either RR08 or both components of TCS08 procured a 2-fold decrease of PavB in both D39 and TIGR4 (Fig. 4). Similarly, the surface abundance of PavB was higher in the *hk08* mutant and lower in the *rr08* and *tcs08* mutants as indicated by flow cytometry (Fig. 5). Importantly, these data fit with the gene expression analysis of the mutants by microarrays.

**FIG 6:**
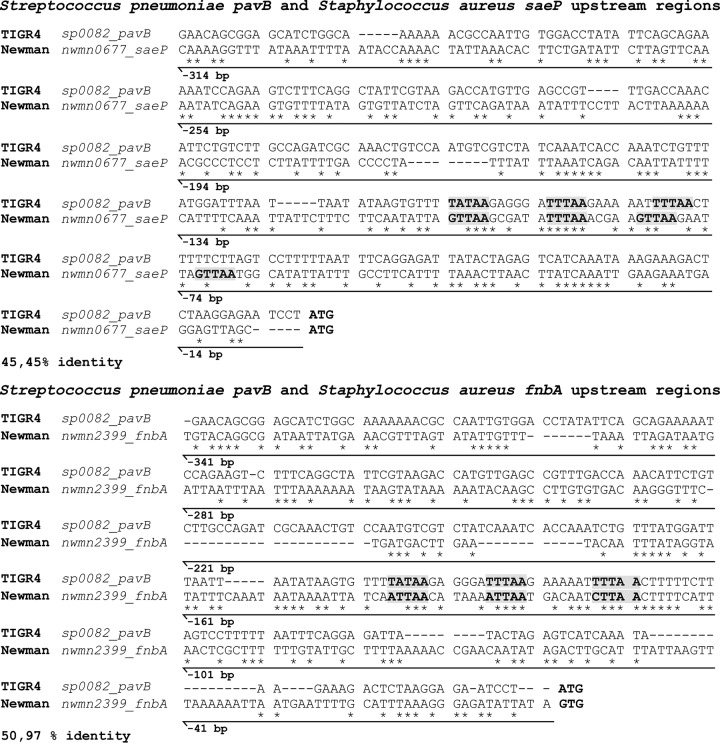
Sequence comparison of upstream regions from the genes *pavB*, *saeP*, and *fnbA*. An *in silico* alignment was performed using 300 bp upstream of the pneumococcal *pavB* and the staphylococcal *saeP* and *fbnA* genes. The arrows indicate the distance upstream from the starting ATG. The bold letters in the gray boxes highlight the SaeR binding motifs in all sequences. The alignment was done using the Clustal Omega tool from the EMBL-EBI. The DNA sequences were retrieved from the Kyoto Encyclopedia for Genes and Genomes (KEGG). Asterisks indicate conserved base pairs.

Furthermore, an *in silico* comparison of a 300-bp upstream region of the pneumococcal gene *pavB* and the staphylococcal *saeP* and *fnbA* genes revealed the presence of a SaeR-like binding motif for *pavB* (Fig. 6). The SaeR-like binding motif is 76 bp upstream of the starting ATG of *pavB* and within its putative promoter region. In conclusion, TCS08 interferes with the regulation of adhesins and may therefore also have an impact on colonization.

### TCS08 modulation of lung infections and sepsis is strain dependent.

**FIG 7:**
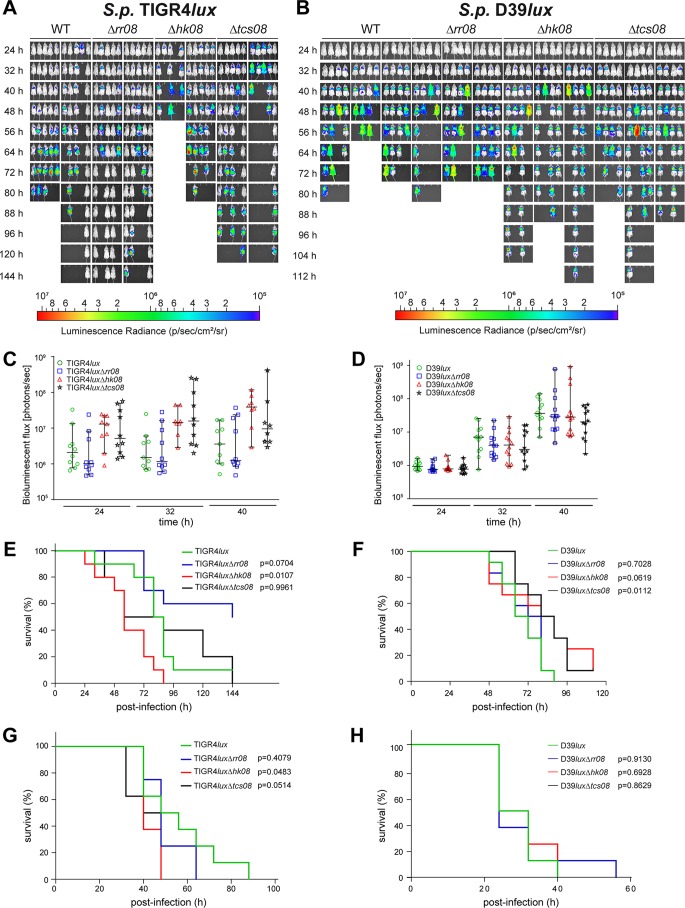
Influence of the TCS08 components on pneumococcal pathogenesis. CD-1 mice were used in the acute pneumonia model to determine the impact of the TCS08 components on virulence. Infection doses of 1 × 10^7^ and 7 × 10^7^ bacteria were applied for *S. pneumoniae* D39 and TIGR4, respectively. (A and B) Bioluminescent (*lux*) strains were used to monitor the progression of the disease *in vivo*. The results are shown as photon flux change (C and D) and analyzed by a Kaplan-Meier plot (E and F). For the sepsis model (G and H), 1 × 10^3^ bacteria were used as the infection dose for both wild-type strains and corresponding mutants. A log rank test was used for the statistical test with a group size of *n* = 12 (D39) or *n* = 10 (TIGR4), and the error bars indicate the standard deviations.

To assess the impact of TCS08 or its individual components on pneumococcal colonization, lung infection, or sepsis, CD-1 mice were intranasally or intraperitoneally infected with bioluminescent wild-type strains (D39 or TIGR4) and corresponding isogenic mutants. In D39, intranasal infections with mutants lacking either HK08 or both components of TCS08 increased the survival time of mice; thus, the mutants were attenuated and represent a less virulent phenotype (Fig. 7B and F). The *rr08* mutant of D39 showed no differences in developing lung infections (Fig. 7B and F). In the sepsis model, no differences between the wild type of D39 and its isogenic mutants were observed (Fig. 7H). Strikingly and in contrast to D39 infections, the acute pneumonia and sepsis infection models indicated a higher virulence potential of TIGR4 bacteria lacking HK08. In contrast, the loss of RR08 in the TIGR4 genetic background resulted in a significantly attenuated phenotype, leading to the survival of 50% of the infected mice. No differences were observed when both components of TCS08 were absent in TIGR4 (Fig. 7A, E, and G).

The impact of TCS08 on colonization and lung infection was further investigated in the competitive mouse infection assay using the intranasal infection route. Interestingly, the wild-type TIGR4 has a lower number of recovered bacteria than the *rr08* mutant, while having a significantly higher number in the nasopharynx or bronchoalveolar lavage fluid compared to the *hk08* mutant 24 and 48 h postinfection (Fig. S2). Taken together, it becomes clear that TCS08 and its individual components are essential for a balanced homeostasis, thereby maintaining pneumococcal fitness and robustness.

10.1128/mSphere.00165-18.1 FIG S1  Generation of pneumococcal *tcs08* mutants. (A) Schematic model of the gene organization and insertion deletion mutagenesis by allelic replacement of the *tcs08* operon in S. pneumoniae TIGR4 as an example for all produced mutants. An *in silico* search for operon conformation identified the transcription start and terminator for the *tcs08* operon as indicated by the black arrow and lollipop, respectively. (B) Mutants were also confirmed by real-time PCR (qPCR). Specific primers were used for *rr08* and *hk08*. Additionally, the ribosomal protein S16 (*sp_0775*) was used as a control. Download FIG S1, PDF file, 0.2 MB. Copyright © 2018 Gómez-Mejia et al. 2018 Gómez-Mejia et al. This content is distributed under the terms of the Creative Commons Attribution 4.0 International license.

10.1128/mSphere.00165-18.2 FIG S2  Coinfection assay with TIGR4 wild type and isogenic *tcs08* mutants. Competition assays between wild type and TCS08 mutants were carried out in *S*. *pneumoniae* TIGR4. CD-1 mice were intranasally inoculated with a mixture of bioluminescent TIGR4 and each *tcs08* mutant with an infection dose of 2.5 × 10^7^ bacteria of each strain. Mice were sacrificed, and the samples were collected after 24 and 48 h. Colony determination data were plotted as the mutant/wild-type ratio to determine the CI for the nasopharyngeal (A) and bronchoalveolar (B) lavages. Results are displayed as scatter plots with each dot representing one mouse and the solid line indicating the median. Download FIG S2, PDF file, 0.1 MB. Copyright © 2018 Gómez-Mejia et al. 2018 Gómez-Mejia et al. This content is distributed under the terms of the Creative Commons Attribution 4.0 International license.

10.1128/mSphere.00165-18.3 FIG S3  Localization, orientation, and grouping of the regulated genes (159 genes found by the microarray study) by the pneumococcal TCS08. Genes under regulation by the pneumococcal TCS08 are illustrated in a linear representation of the genome of Streptococcus pneumoniae. The left panel indicates the localization and orientation of each gene, where localization on the positive strand is indicated by red and that on the negative strand is indicated in blue. The right panel of the figure groups the genes in 5 different biochemical categories according to the characterization suggested by the databases KEGG (Kyoto Encyclopedia of Genes and Genomes) and BacMap (Bacterial Map genome atlas): green indicates environmental information processing (EIP), yellow indicates intermediary metabolism (IM), blue indicates colonization factors (CF), pink indicates genetic information processing (GIP), and gray indicates genes of unknown function (UF). Download FIG S3, PDF file, 0.1 MB. Copyright © 2018 Gómez-Mejia et al. 2018 Gómez-Mejia et al. This content is distributed under the terms of the Creative Commons Attribution 4.0 International license.

## DISCUSSION

The role of a subset of pneumococcal TCS in competence, physiology, and virulence has been characterized, providing an initial understanding of their specific regulons (10, 12, 37). As such, TCS08 of S. pneumoniae has been initially identified and suggested to be important for virulence (11, 12, 37). Nevertheless, the mechanism underlying its effect on pathophysiological processes has not been elucidated before. A valid approach to estimate the regulons and effects of a TCS is to analyze the protein structures of its components. Unfortunately, only the structure of the pneumococcal RR11 and RR14 has been solved experimentally (38, 39). Nevertheless, it is possible to estimate the likely structural disposition of the remaining components by using bioinformatic tools. As such, according to the information obtained by the database SMART (Simpler Modular Architecture Research Tool, http://smart.embl-heidelberg.de/), the pneumococcal histidine kinase 08 can be classified as an intermembrane histidine kinase (IM-HK) due to its short extracellular loop. Members of this class of HKs are known to respond to membrane disturbances (26). Additionally, RR08 is classified as a member of the OmpR class of response regulators, known to bind to short tandem repeats of DNA (40). Both components share high homology and similar sequence features with the HK SaeS and RR SaeR from S. aureus (Fig. S4) (24). Altogether, it is plausible to hypothesize that the regulatory behavior of the pneumococcal TCS08 is similar to the global virulence regulatory system SaeRS of Staphylococcus aureus.

10.1128/mSphere.00165-18.4 FIG S4  Staphylococcal SaeRS and pneumococcal TCS08 sequence alignment. Amino acid sequence alignment between the histidine kinases and response regulators of the pneumococcal TCS08 and the staphylococcal SaeRS systems. The red residues indicate the reported histidine and aspartate residues for SaeS and SaeR, respectively. The sequence comparison was performed using the Clustal Omega tool from the EMBL-EBI, and the protein sequences were retrieved using the Kyoto Encyclopedia of Genes and Genomes (KEGG). The asterisk indicates a score of 1, the colon indicates a score of >0.5, and the single period indicates a score of >0 and <0.5. Download FIG S4, PDF file, 0.3 MB. Copyright © 2018 Gómez-Mejia et al. 2018 Gómez-Mejia et al. This content is distributed under the terms of the Creative Commons Attribution 4.0 International license.

The staphylococcal SaeRS TCS is known to be essential for the virulence of S. aureus by regulating approximately 20 virulence genes such as the α-hemolysin gene (*hla*), the fibronectin binding protein A gene (*fbnA*), and its own SaePQRS operon, among others (27). However, there are only a few reports regarding the control of staphylococcal fitness by the SaeRS system. One study investigated a negative regulatory effect of fatty acids on the phosphorylation of SaeS and the activation of the virulence factors controlled by SaeR (41). Our initial approach to investigate the regulatory roles of the pneumococcal TCS08 by transcriptomics discovered five main gene categories influenced by this TCS. Interestingly, we observed the most predominant regulation for genes participating in environmental information processing and intermediary metabolism (Fig. 2; Table S1). The genes grouped in these two categories are annotated as part of ABC transporters, phosphotransferase systems, and lipid biosynthesis and were found to be localized all along the pneumococcal genome (Fig. S3). The genes found to be regulated by TCS08 share an important feature, namely, their localization and/or activity in the pneumococcal membrane. Additionally, several of the different PTS and ABC transporters regulated by TCS08 are involved in the fitness and virulence of this pathogen. Hence, the effect of TCS08 is more pronounced in the colonization phase of the pneumococcal life cycle. This is, for example, the case for the neuraminidase NanA, lipoprotein PsaA, and arginine deiminase system (ADS) (31, 42–44). Moreover, the observed regulation of the complete *fab* operon encoding enzymes for fatty acid biosynthesis creates an important connection between TCS08 and sensing and responding to membrane instability (19, 45). The transporter systems affected by TCS08 are mostly essential during colonization under nutrient-limiting conditions but also in the initial stages of the diseases to take up nutrients and ensure pneumococcal fitness (Fig. 2 and 3) (46, 47).

In addition to the gene expression analysis of *tcs08* mutants, we further investigated the changes on the protein level for selected candidate proteins. Our immunoblot analyses demonstrated differences for PsaA and the arginine deiminase ArcA. Remarkably, compared to the respective wild-type strains ArcA occurred at higher protein levels in all mutants of D39 and the TIGR4 mutant lacking both HK08 and RR08 (2-fold), while ArcA had lower protein levels in the TIGR4 mutants lacking either HK08 or RR08 (2-fold). However, only the opposite effect of deletion of *hk08* on the ArcA level was statistically significant. This is a further proof that the ADS in D39 and TIGR4 is differentially regulated, as has been shown before for the stand-alone regulator ArgR2. There, the *arc* operon showed a constitutive expression in D39, while in TIGR4 gene expression was upregulated by ArgR2 (31).

It is essential that pneumococci activate their metabolic inventory when colonizing their host to ensure adaptation and fitness. As such, our results point to a role of TCS08 in the fine-tuning of colonization and metabolic homeostasis as exemplified by the level of change in the expression of *pavB* and the genes of the *pilus-1*, *fab*, and *arc* operons.

*pavB* belongs to a group of genes regulated by TCS08 which are strongly involved in colonization by its interactions with host proteins (32, 33). This group of genes codes mostly for surface-exposed proteins associated with peptidoglycan metabolism and adherence to host cells. These genes are found grouped clockwise mostly in the first quarter of the pneumococcal genome, and transcription and replication proceed into the same direction (Fig. S3). Interestingly, the regulation of the adhesin PI-1 and PavB proteins by the pneumococcal TCS08 illustrates the high homology between the staphylococcal SaeRS and pneumococcal TCS08. Differences in gene expression of the PI-1 component genes were detected by microarray analysis (Fig. 2) and qPCR (Fig. 3) in the TIGR4 TCS08 mutants. Similarly, protein levels were also affected in the TCS08 mutants, especially in the absence of both components of TCS08, in which strong downregulation was detected (Fig. 4A and 5A). Our findings correlate to some extent with a previous study showing the regulation of PI-1 by the pneumococcal TCS08 (28). For the adhesin PavB, inconsistent results were obtained for gene expression and protein abundance in the D39 strain. A minor but significant differential *pavB* gene expression was measured by microarray analysis and qPCR for TIGR4 (Fig. 2 and 3). In contrast, PavB protein levels were significantly affected in all mutants, with a 2-fold increase in the absence of HK08 and a decrease in PavB in mutants lacking either RR08 or both components of TCS08 as shown by immunoblotting and flow cytometry (Fig. 4 and 5).

The staphylococcal fibronectin binding protein FbnA is weakly regulated by the SaeRS system of S. aureus (48), which in pneumococci correlates with the link found between TCS08 and PavB/PI-1. A direct repeat sequence (TTTAAN_7_TTTAA), similar to the imperfect SaeR binding site (GTTAAN_6_TTTAA) (25), can be found directly upstream of *pavB* (Fig. 6), suggesting that RR08 binds directly to the *pavB* promoter region. A strong hint for *pavB* gene regulation by TCS08 is the higher abundance of PavB in the absence of HK08. Surprisingly, a conserved repeat sequence, TTTAAN_14_GTTAA, was found close to the *rlrA* operon and could indicate an indirect effect of TCS08 in the regulation of *pilus-1* via its positive regulator RlrA (Table S5). The *in silico* search for SaeR-like binding motifs among different TCS08-regulated genes indicated the presence of a variation of this binding sequence for the cellobiose and *arc* operons, while it was absent for the *psa* operon (Table S5). All of the genes carried in these operons have been reported to be under the regulation of CcpA-dependent stand-alone regulators (31, 45, 49, 50). Additionally, the *psa* operon has been also shown to be under the regulation of PsaR and TCS04 (PnpRS), which might be interplaying with TCS08 (51, 52). This suggests either a cooperative role or a collateral effect of TCS08, and we hypothesize that TCS08 acts as a membrane stability sensor system.

The staphylococcal SaeRS was further reported to regulate proteases and be involved in biofilm formation. Our microarray analysis showed an effect on the expression for genes encoding a putative protease domain (Fig. 2 and 3) such as the gene (*sp_0144*) possessing an Abi (abortive infective domain) with unknown function in pneumococci. Bioinformatic analysis revealed that pneumococcal *sp_0144* is highly homologous to *spdABC* genes of S. aureus Newman, featuring an Abi domain. Interestingly, the SpdA, SpdB, and SpdC proteins have been reported to be involved in the deposition and surface abundance of sortase-anchored proteins in S. aureus (53). The gene expression of *sp_0144* (TIGR4) presented an upregulation in the *hk08* mutant in TIGR4. It cannot be ruled out that the changes in SP_0144 also contribute to the protein abundance demonstrated for PavB or PI-1 when the strains lack components of TCS08 (Fig. 3). In turn, changes in surface abundance of colonization factors will interfere with the pneumococcal virulence and/or immune evasion. However, this hypothesis was not evaluated in this study and needs experimental proof in a follow-up study.

Nasopharyngeal colonization by pneumococci requires adherence to host cells and generates a foothold in the human host. Hence, the regulation of adhesins and ECM binding proteins like PavB or PI-1 represents a successful strategy of the pathogen to adapt to this host compartment. Similarly, the sensing of human neutrophil peptides and membrane disruption molecules is also essential to ensure a successful colonization and immune escape phenotype. Our *in vivo* studies using pneumonia and sepsis murine models confirmed the contribution of the pneumococcal TCS08 not only in colonization but also virulence (Fig. 7). However, the effect is strain dependent, highlighting the role and network of different stand-alone regulators and other regulatory systems of pneumococci in the overall regulation of pneumococcal fitness and pathophysiology. Such strain-dependent effects have been also shown for additional pneumococcal TCS such as PnpRS and TCS09 (ZmpRS) (51, 54). Remarkably, a more virulent phenotype was observed for the TIGR4 mutant lacking HK08, while the TIGR4 mutant deficient for RR08 displayed a decrease in virulence in the pneumonia model (Fig. 7A). In D39, the opposite effect with a slight increase in survival was observed in the absence of HK08 in the same infection model (Fig. 7B). Additionally, the loss of function of both TCS08 components in strain D39 resulted in a significant reduction in virulence in the pneumonia model (Fig. 7B). Strikingly, this D39 attenuation was not observed in the sepsis model. Similarly, the TIGR4 *rr08* mutant was also as virulent as the wild type, despite being attenuated in the pneumonia model (Fig. 7G and H). In contrast, the TIGR4 Δ*hk08* mutant was significantly more virulent than the wild type in the sepsis model (Fig. 7G). As such, our results suggest that TCS08 is mostly involved in bacterial fitness and regulation of adhesins required for a successful colonization. Such striking differences between two representative pneumococcal strains may reflect their different genomic background and the overall versatility of pneumococci.

Interesting pathophenotypes were observed in competitive mouse infections, i.e., coinfections with the TIGR4 wild type and its *tcs08* isogenic mutants (Fig. S2). While the pneumonia model showed an avirulent phenotype in the absence of RR08, this mutant revealed a higher competitive index (CI) than its wild type in the coinfection assay in both the nasopharyngeal and bronchoalveolar lavage fluids, indicating lower numbers of the wild type in these host compartments. In addition, TIGR4 mutants lacking either HK08 or both components of TCS08 were apparently outcompeted by the wild type (Fig. S2) despite being more virulent than the wild type as indicated in the acute pneumonia model. A plausible explanation for this phenomenon might be that the TIGR4 mutant lacking HK08 is rapidly progressing from the nasopharynx and lungs into the blood and, hence, low numbers are present in the nasopharynx and lavage. Similarly, the absence of RR08 impairs progressing into the blood, and thus, higher numbers of the *rr08* mutant are found in the nasopharynx. Indeed, this pneumococcal behavior after nasopharyngeal infection can also be visualized in the bioluminescent images of the acute pneumonia model, in which the mice infected with the strain lacking HK08 rapidly developed pneumonia and sepsis (Fig. 7A).

It is also important to mention here the mild impact of TCS08 on gene expression alterations. This suggests a role for TCS08 as a fine-tuning and signal modulation system, which is dependent on additional regulators. This hypothesis is supported by the altered gene expression of other TCS such as CiaRH and ComDE (Fig. 2; Table S1). Such low impact on gene expression might also facilitate an explanation for the predominant role of HK08 in controlling gene expression in pneumococci. A similar regulatory strategy has been reported for CiaRH. This system is able to control directly the expression of the protease HtrA and specific small RNAs, which in turn modulate indirectly the activity of ComDE and additional regulators (55, 56). We therefore hypothesize that the stimulus received by HK08 modulates the activity of RR08 and probably other regulators. In Staphylococcus aureus, the SaeRS system is also dependent on additional auxiliary proteins SaePQ (57). These proteins have been reported to interact with SaeS in order to control its phosphorylation state (57). Such systems have not yet been detected for the homologous TCS08 of the pneumococci. However, a more thorough biochemical analysis would be needed to generate a comprehensive regulatory map within pneumococcal regulators.

In conclusion, this study identified five main groups of genes influenced by the pneumococcal TCS08 in a strain-specific manner. A high number of these genes encode proteins involved in environmental signal processing, intermediary metabolism, colonization, or genetic information processing. Furthermore, most of the TCS08-regulated proteins are membrane bound and involved in nutrient transport as well as fatty acid biosynthesis. Additionally, surface-exposed PavB and PI-1 islet proteins involved in adhesion to host components were confirmed to be controlled by TCS08. Thus, HK08 of TCS08 is probably sensing small molecules entering the membrane compartment of pneumococci and adapts thereby the pneumococcus to the specific environmental conditions during colonization.

## MATERIALS AND METHODS

### Bacterial strains and growth conditions.

S. pneumoniae and Escherichia coli strains used in this study are listed in Table S2 in the supplemental material. Pneumococcal wild type and isogenic *tcs08* deletion mutants were grown on Columbia blood agar plates (Oxoid) containing selection antibiotics (kanamycin [Km; 50 µg/ml] and erythromycin [5 µg/ml] or spectinomycin [Spec; 100 µg/ml]) using an incubator at 37°C, 5% CO_2_. In liquid cultures, pneumococci were cultivated in Todd-Hewitt broth (Roth) supplemented with 0.5% yeast extract (THY) or chemically defined medium (CDM; RPMI 1640 plus 2 mM l-glutamine medium [HyClone GE Healthcare Life Sciences] supplemented with 30.5 mM glucose, 0.65 mM uracil, 0.27 mM adenine, 1.1 mM glycine, 0.24 mM choline chloride, 1.7 mM NaH_2_PO_4_⋅H_2_O, 3.8 mM Na_2_HPO_4_, and 27 mM NaHCO_3_) using a water bath at 37°C. Recombinant E. coli strains were inoculated on lysogeny broth (LB) medium (Roth) in the presence of kanamycin (Km; 50 µg/ml) at 37°C using an orbital shaker.

10.1128/mSphere.00165-18.6 TABLE S2  List of strains and mutants. Download TABLE S2, PDF file, 0.1 MB. Copyright © 2018 Gómez-Mejia et al. 2018 Gómez-Mejia et al. This content is distributed under the terms of the Creative Commons Attribution 4.0 International license.

### Molecular techniques.

The oligonucleotides and plasmid constructs used in this study are listed in Tables S3 and S4. The isolation of pneumococcal chromosomal DNA was achieved by using the standard phenol-chloroform extraction protocol. Briefly, S. pneumoniae strains were cultured in blood agar for 6 h, transferred to new blood agar plates with antibiotics, and grown for 10 h at 37°C and 5% CO_2_. After inoculation in THY liquid medium and culture until reaching an optical density at 600 nm (OD_600_) of 0.6 in a water bath at 37°C, the bacteria were harvested by centrifugation. The supernatant was discarded, and the bacterial pellet was resuspended in Tris-EDTA sodium (TES) buffer for lysis and processing. Finally, the DNA was extracted using phenol and phenol-chloroform-isoamyl alcohol (25:24:1), washed with 96% ethanol, and stored in Tris-EDTA (TE) buffer at −20°C for further use. The DNA regions needed for mutant generation and for protein production were amplified by PCR using the *Pfu* proofreading polymerase (Stratagene, La Jolla, CA, USA) and specific primers (Eurofins MWG Operon, Germany) according to the manufacturer’s instructions. The annealing and extension temperatures were defined by the primers and length of the DNA inserts, respectively. The PCR products and the plasmids were purified using the Wizard SV gel and PCR cleanup system (Promega, USA). The final constructs were confirmed by sequencing (Eurofins MWG).

10.1128/mSphere.00165-18.7 TABLE S3  List of primers. Download TABLE S3, PDF file, 0.1 MB. Copyright © 2018 Gómez-Mejia et al. 2018 Gómez-Mejia et al. This content is distributed under the terms of the Creative Commons Attribution 4.0 International license.

10.1128/mSphere.00165-18.8 TABLE S4  List of plasmids. Download TABLE S4, PDF file, 0.03 MB. Copyright © 2018 Gómez-Mejia et al. 2018 Gómez-Mejia et al. This content is distributed under the terms of the Creative Commons Attribution 4.0 International license.

10.1128/mSphere.00165-18.9 TABLE S5  *In silico* search for RR08 binding motifs. Download TABLE S5, PDF file, 0.1 MB. Copyright © 2018 Gómez-Mejia et al. 2018 Gómez-Mejia et al. This content is distributed under the terms of the Creative Commons Attribution 4.0 International license.

### S. pneumoniae mutant generation.

For mutant generation in D39 and TIGR4 (Δ*cps* and bioluminescent [*lux*] strains), the insertion-deletion strategy was applied by amplifying 5′ and 3′ flanking regions of *rr08*, *hk08*, and the full *rr08-hk08* operon via PCR with specific primers. The genomic fragments were cloned in a pGEM-T Easy vector, transformed into E. coli DH5α, and further processed by inverse PCR using primers to delete the desired target gene and replacing it with either spectinomycin (*aad9*) or erythromycin (Erm^r^) resistance gene cassettes. To achieve the deletion of the desired regions, the inverse PCR products and antibiotic cassettes were digested using specific restriction enzymes (Table S3). Finally, the deleted gene fragments encompass the following regions in each mutant: bp 29 to 953 in Δ*hk08*, bp 100 to 644 in Δ*rr08*, and bp 128 of *rr08* to bp 348 of *hk08* in Δ*tcs08*. Pneumococcal strains were transformed as described previously (31; see also ref. 60) using competence-stimulating peptide 1 (CSP1) (D39) or CSP2 (TIGR4) and cultivated in the presence of the appropriate antibiotics: kanamycin (50 µg/ml) and erythromycin (5 µg/ml) or spectinomycin (10 µg/ml). Briefly, S. pneumoniae strains were cultured on blood agar plates for 8 h, and a second passage was done for 10 h in an incubator at 37°C and 5% CO_2_. Later, the strains were inoculated in THY with an initial OD_600_ of 0.05 and grown in a water bath until reaching a final OD_600_ of 0.1. The corresponding CSP was added and incubated at 37°C for 15 min, followed by the addition of the plasmid for transformation and a heat shock treatment of 10 min on ice and 30 min at 30°C. Bacteria were allowed to grow for 2 h at 37°C and plated on blood agar plates with the corresponding antibiotics. The resulting S. pneumoniae D39 and TIGR4 *tcs08*-deficient mutants were screened by colony PCR and real-time PCR (qPCR) (Fig. S1B). Stocks were generated in THY supplemented with 20% glycerol and stored at −80°C. Individual mutants for *rr08* (*sp_0083*) and *hk08* (*sp_0084*) as well as a Δ*tcs08* (*sp_0083* + *sp_0084*) mutant were confirmed by colony PCR.

### Transcription analysis by microarrays.

For the analysis of the gene expression by microarray, TIGR4 Δ*cps* and its isogenic *rr*, *hk*, and *tcs08* mutants were grown in CDM until reaching an OD_600_ of 0.35 to 0.4 in triplicate. Bacterial cultures were then added to previously prepared tubes containing frozen killing buffer (20 mM Tris-HCl [pH 7.5], 5 mM MgCl_2_, 20 mM NaN_3_) and centrifuged for 5 min at 10,000 × *g*. The supernatant was completely removed, and the tubes containing the pellets were immediately flash frozen in liquid nitrogen and stored at −80°C until the next step. The pellets were processed for total RNA extraction using acid phenol-chloroform and DNase treatment to remove genomic DNA. The products were purified using the RNA cleanup and concentration kit (Norgen Biotek Corp.), the quality of the RNA was determined with an Agilent 2100 Bioanalyzer, and the amount was quantified using a NanoDrop ND-1000 spectrophotometer (Peqlab). Five micrograms of total RNA was subjected to cDNA synthesis as described by Winter et al. (58). One hundred nanograms of Cy3-labeled cDNA was hybridized to the microarray according to Agilent’s hybridization, washing, and scanning protocol (One-Color microarray-based gene expression analysis, version 6.9.1). Data were extracted and processed using the Feature Extraction software (version 11.5.1.1). Further data analysis was performed using the GeneSpring software (version 14.8). A Student *t* test with a *P* value of <0.05, followed by a Benjamini and Hochberg false discovery rate correction with a *q* value of <0.05, was performed for the analysis.

### Gene expression analysis by qPCR.

D39 and TIGR4 Δ*cps* strains and their corresponding *tcs08* mutants were grown in triplicate in CDM until early log phase and harvested for RNA isolation using the EURx GeneMatrix universal RNA purification kit (Roboklon). The RNA was checked for quality and contamination by PCR and agarose gel electrophoresis. Next, cDNA synthesis was carried out using the Superscript III reverse transcriptase (Thermo Fisher) and random hexamer primers (Bio-Rad). The obtained cDNA was checked by PCR using the same specific primers designed for the qPCR studies (Table S3). The cDNA was measured by a NanoDrop spectrophotometer and stored at −20°C until further tests. For the qPCR experiments, a StepOnePlus thermocycler (Applied Biosystems) with a Syber Green master mix (Bio-Rad) was used according to the instructions for relative quantification to determine the efficiency of the primers, and as such, a reference curve was designed to be run for every gene with 5 points and concentrations ranging from 100 ng/µl to 0.01 ng/µl with 1:10 dilution steps. The StepOne software (version 2.3; Life Technologies) and Microsoft Office Excel 2016 software (Microsoft) were used for the analysis. The final results are plotted as the threshold cycle (ΔΔ*C*_*T*_) (log_2_ of the fold change of expression), with the wild-type value set to 0 and compared to its respective *tcs08* mutants. For normalization, the gene encoding the ribosomal protein S16 (*sp_0775*) was used. The results are plotted as box-whisker plots showing the median and 95% confidence intervals and as a heat map.

### Protein expression by immunoblotting.

S. pneumoniae D39 and TIGR4 strains and their isogenic mutants were grown in CDM, harvested at mid-log phase, and resuspended in phosphate-buffered saline buffer (PBS). A total of 2 × 10^8^ cells were loaded and run on a 12% SDS-PAGE gel and further transferred into a nitrocellulose membrane. Mouse polyclonal antibodies generated against different pneumococcal proteins and a secondary fluorescence-labeled IRDye 800CW goat anti-mouse IgG antibody (1:15,000) were used to detect their expression in the wild type (WT) and its isogenic mutants using the Odyssey CLx scanner (Li-Cor). Rabbit polyclonal antibody against enolase (1:25,000) and fluorescence-labeled IRDye 680RD goat anti-rabbit IgG antibody (1:15,000) were used as loading controls for normalization. The quantification was performed using Image Studio software (Li-Cor), and the data are presented as the log_2_ of the fold change with the wild-type value set to 0 and compared to each mutant after normalization against enolase. The Student *t* test was used for the statistical analysis.

### Surface abundance of proteins analyzed by flow cytometry.

The expression and abundance of different surface proteins were analyzed by flow cytometry. To detect the antigens, specific primary antibodies were used in conjunction with fluorescence-tagged secondary antibodies. In brief, nonencapsulated bacteria (D39 Δ*cps* and TIGR4 Δ*cps*) and the isogenic *tcs08* mutants were used after growth in CDM until reaching a final OD_600_ of 0.35 to 0.4. Bacteria were washed with 5 ml PBS and finally resuspended in 1 ml PBS supplemented with 0.5% fetal calf serum (FCS). The bacterial cell density was adjusted to 1 × 10^7^ cells/ml in 1 ml of PBS-0.5% FCS-1% paraformaldehyde (PFA), loaded into a 96-well microtiter plate (U bottom), and incubated for 1 h at 4°C. The plates were centrifuged at 3,200 × *g* for 6 min, the supernatant was removed, and bacteria were incubated for 45 min at 4°C with antigen-specific mouse antibodies (31, 32, 59). Samples were washed twice with PBS-0.5% FCS and incubated with goat anti-mouse Alexa 488 (1/1,000 dilution) antibody for 45 min. Thereafter, the plate was washed twice with PBS-0.5% FCS and fixed using 1% PFA in the dark at 4°C overnight (o/n). Fluorescence of the bacteria was measured using a BD FACSCalibur machine equipped with log forward and log side scatter plots. The measurement of the data was conducted with CellQuestPro software 6.0. (BD Biosciences), collecting 50,000 events and a gated region. The results were analyzed using the freeware Flowing software version 2.5.1 (Turku Centre for Biotechnology, University of Turku, Finland) and presented as the geometric mean fluorescence intensity (GMFI) of the analyzed bacterial population by the percentage of labeled bacteria.

### Impact of TCS08 in murine pneumonia and sepsis models.

Bioluminescence-expressing S. pneumoniae D39 *lux* and TIGR4 *lux* and their isogenic mutants were grown in THY supplemented with 10% heat-inactivated fetal calf serum (FCS) until reaching an OD_600_ of 0.35 to 0.4 and harvested via centrifugation at 3,270 × *g* for 6 min. The bacteria were resuspended in PBS, and the CFU were adjusted for an infection dose of 1 × 10^7^ CFU in 10 µl or 5 × 10^3^ CFU in 200 µl per mouse for the pneumonia and sepsis models, respectively. The infection process for pneumonia was carried out as follows: 8- to 10-week-old 10 to 12 CD-1 outbred mice were arranged in groups of 5 or 4 animals per cage, respectively, and anesthetized with an intraperitoneal injection of 200 µl of ketamine 10% (mg/ml) and 2% xylazine (Rompun) (dose is determined according to the weight of the animals). The mice were held facing upward, and 20 µl of infection dose (10 µl bacteria plus 10 µl hyaluronidase [90 U]) was pipetted carefully in the nostrils. Mice were allowed to inhale the drops and rest facing upward until the anesthesia wore off. The infection dose was controlled by plating in triplicate dilutions of the bacterial solution on blood agar plates and counting the colonies. The infection was monitored in real time using the IVIS Spectrum system and imaging software. Mice were monitored after the first 24 h and every 8 h from then on until the end of the experiment. For the sepsis model, 8- to 10-week-old CD-1 outbred mice (*n* = 8) were arranged in groups of 4 animals per cage and intraperitoneally infected with 200 µl containing 5 × 10^3^ CFU. Mice were monitored 16 h postinfection and every 8 h from then on until the end of the experiment. The infection dose was confirmed by plating different dilutions of the infection dose. The results were annotated using GraphPad Prism version 7.02 software and presented in a Kaplan-Meier (KM) graph. The log rank test was used for the statistics.

Bioluminescent TIGR4 wild type and its corresponding *tcs08* mutants were applied in the coinfection assay. Briefly, an infection dose of 2.5 × 10^7^ CFU of wild type and a single mutant (Δ*rr08*, Δ*hk08*, or Δ*tcs08*) was mixed (1:1 ratio) and mice (*n* = 10 CD-1) were intranasally infected. The infection dose was determined by plating serial dilutions of the infection mixture onto plates with kanamycin or kanamycin plus erythromycin/Spec to enumerate CFU of the wild type and mutant or CFU of the mutant. Mice were sacrificed after 24 and 48 h, and nasopharyngeal and bronchoalveolar lavages were performed using a tracheal cannula filled with 1 ml of sterile PBS. The recovered solution was diluted and plated on blood agar plates with appropriate antibiotics (see above). Colonies were counted, and recovered CFU of the wild type and mutant was determined. The competitive index (CI) was calculated as the mutant/wild-type ratio. Values higher than 1 indicate a higher ratio of mutant bacteria, while values below 1 indicate a higher ratio of wild-type bacteria. The results were annotated using GraphPad Prism version 7.02 software and presented as scatter plots where every dot indicates 1 mouse.

### Ethics statement.

All animal experiments were conducted according to the German regulations of the Society for Laboratory Animal Science (GV-SOLAS) and the European Health Law of the Federation of Laboratory Animal Science Associations (FELASA). All experiments were approved by the Landesamt für Landwirtschaft, Lebensmittelsicherheit und Fischerei, Mecklenburg-Vorpommern (LALLFV M-V, Rostock, Germany, permit no. 7221.3-1-056/16).

### Accession number(s).

Data obtained from the microarray analysis have been uploaded to the National Center for Biotechnology Information (NCBI) at the Gene Expression Omnibus (GEO) ArrayExpress databases (https://www.ncbi.nlm.nih.gov/geo) under accession number GSE108874.
