# Supplementary figures and images for: Pneumococcal Metabolic Adaptation and Colonization Are Regulated by the Two-Component Regulatory System 08
Source: mSphere. 2018 May 16;3(3):e00165-18. doi: 10.1128/mSphere.00165-18 (PMC5956151; doi:10.1128/mSphere.00165-18)

Figure S1

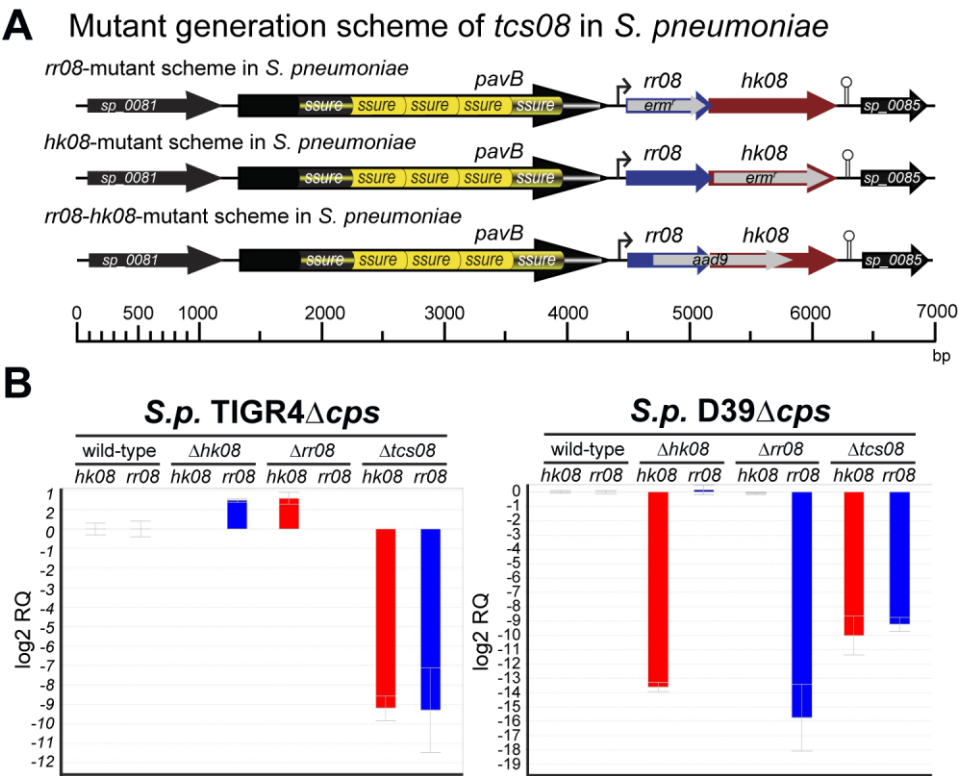

Supplement: FIG S1 [file sph003182549sf1.pdf]

Figure S2

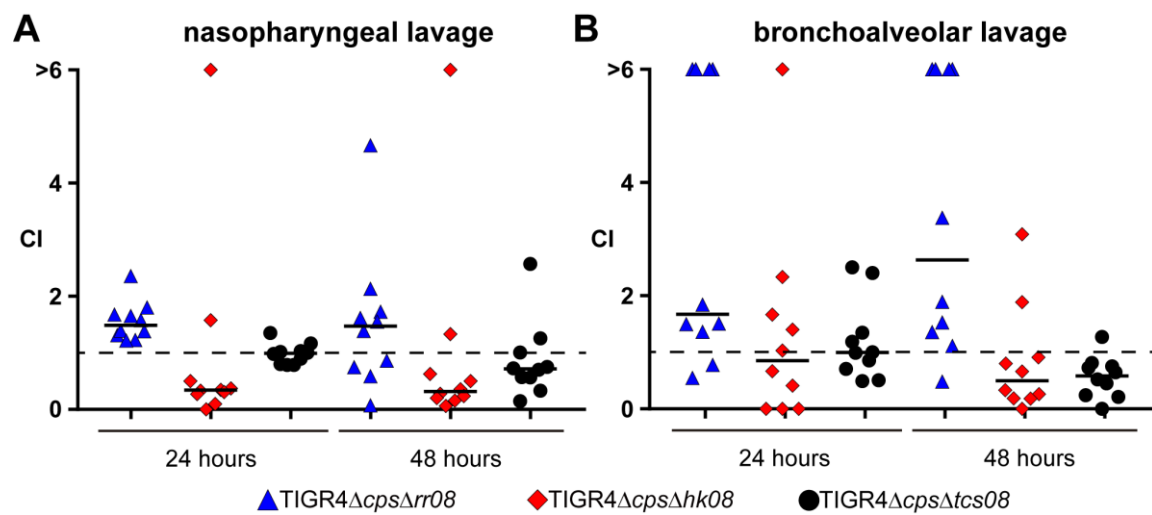

Supplement: FIG S2 [file sph003182549sf2.pdf]

Figure S3

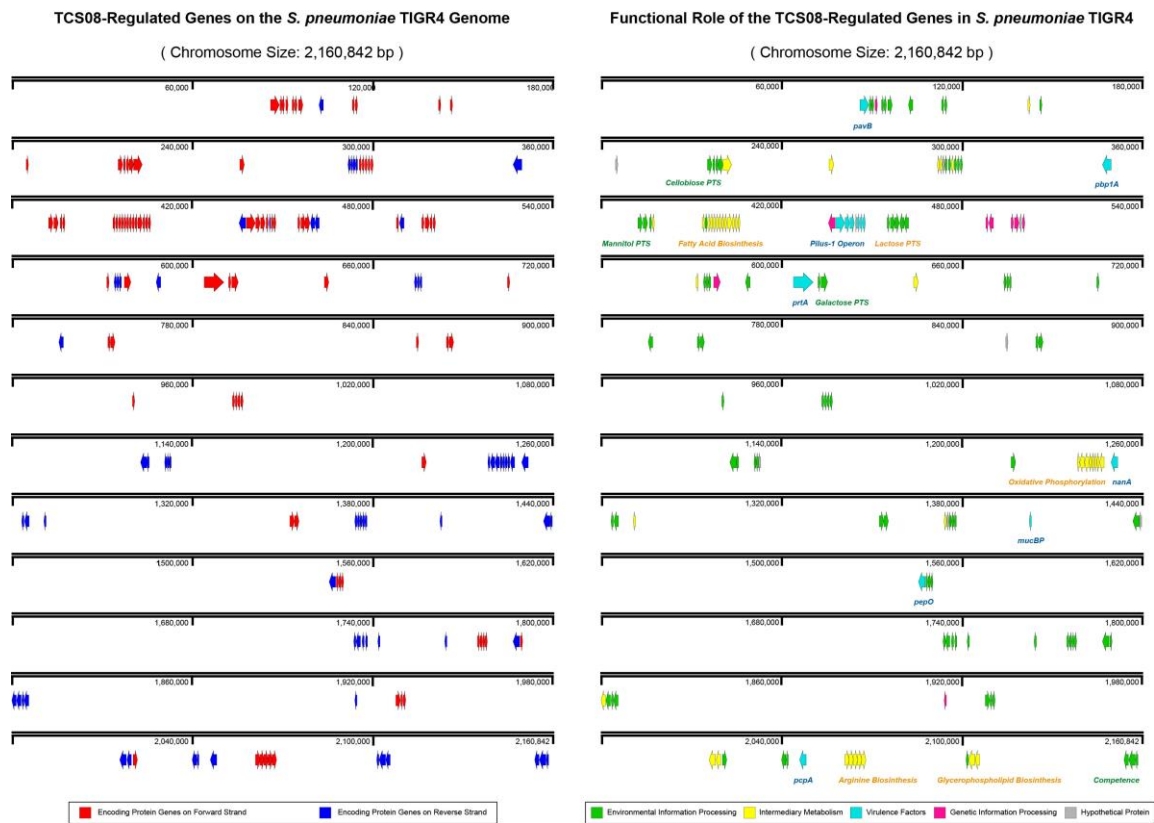

Supplement: FIG S3 [file sph003182549sf3.pdf]
