# Supplementary material for: Pneumococcal Metabolic Adaptation and Colonization Are Regulated by the Two-Component Regulatory System 08
Source: mSphere. 2018 May 16;3(3):e00165-18. doi: 10.1128/mSphere.00165-18 (PMC5956151; doi:10.1128/mSphere.00165-18)
Supplement: FIG S4 [file sph003182549sf4.pdf]

Figure S4

***Streptococcus pneumoniae* HK08 and *Staphylococcus aureus* SaeS protein sequence alignment**

```
TIGR4   SP0084_HK08        --MKLKSYIL VGYIISTLLT ------ILVV FWAVQKMLIA KGEIYFLLGM TIVASLVGAG 60 aa
Newman  NWMN0674_SaeS      MVLSIRSQII IGVVSSIPLT STILAIAYIL MWFNGHMTLT LT------LT TIITSCLTLL
                             :.::* *: :* : *  **         :: :*   :* ::            **::* :
TIGR4   SP0084_HK08        ISLFLLLPVF TSLGKLKEHA KRVAAKDFPS N-LEVQGPVE FQQLGQTFNE MSHDLQVSFD 120 aa
Newman  NWMN0674_SaeS      ICSIFINPLI QKIKQFNIKT KQFANGNYAS NDKTFNSPKE IYELNQSFNK MASEITQQMN
                           *. ::: *::  .: ::: :: *:.*  :: * *   .:.* * : :*.*:**: *: ::  .::
TIGR4   SP0084_HK08        SLEESEREKG LMIAQLSHDI KTPITSIQAT VEGILDGIIK ESEQAHY-LA TIGRQTERLN 180 aa
Newman  NWMN0674_SaeS      QIKSEQQEKT ELIQNLAHDL KTPLASIISY SEGLRDGIIT KDHEIKESYD ILIKQANRLS
                           .::..::**   :* :*:**: ***::** :   **: ****. :..: :      : :*::**.
TIGR4   SP0084_HK08        KLVEELNF-L TLNTARNQVE TTSKDSIFLD KLLIECMSEF QFLIEQERRD VHLQVIPESA 240 aa
Newman  NWMN0674_SaeS      TLFDDMTHII TLNTG----K TYPPELIQLD QLLVSILQPY EQRIKHENRT LEVNFCNEID
                           .*.:::.. : ****.    : *   : * ** :**:. :. : :  *::*.*  :.::.  *
TIGR4   SP0084_HK08        RIEGDYAKLS RILVNLVDNA FKYSAPGTKL EVVA--KLEK DQLSISVTDE GQGIAPEDLE 300 aa
Newman  NWMN0674_SaeS      AFYQYRTPLE RILTNLLDNA LKFSNVGSRI DINISENEDQ DTIDIAISDE GIGIIPELQE
                            :    : *. ***.**:*** :*:*  *::: ::    : :: * :.*:::** * ** **  *
TIGR4   SP0084_HK08        NIFKRLYRVE TSRNMKTGGH GLGLAIAREL AHQLGGEITV SSQYGLGSTF TLVLNLSGSE 360 aa
Newman  NWMN0674_SaeS      RIFERTFRVE NSRNTKTGGS GLGLYIANEL AQQNNAKISV SSDIDVGTTM TVTLHKLDIT
                           .**:* :*** .*** ****  **** **.** *:* ..:*:* **: .:*:*: *:.*:  .
TIGR4   SP0084_HK08        NKA                                                             363 aa
Newman  NWMN0674_SaeS      S--
                           .
```

***Streptococcus pneumoniae* RR08 and *Staphylococcus aureus* SaeR protein sequence alignment**

```
TIGR4   SP0083_RR08        MGKTILLVDD EVEITDIHQR YLIQAGYQVL VAHDGLEALE LFKKKPIDLI ITDVMMPRMD 60 aa
Newman  NWMN0675_SaeR      -MTHLLIVDD EQDIVDICQT YFEYEGYKVT TTTSGKEAIS LLS-NDIDIM VLDIMMPEVN
                             . :*:*** * :*.** *  *:   **:*  .: .* **:. *:. : **:: : *:***.::
TIGR4   SP0083_RR08        GYDLISEVQY LSPEQPFLFI TAKTSEQDKI YGLSLGADDF IAKPFSPREL VLRVHNILRR 120 aa
Newman  NWMN0675_SaeR      GYDIVKEMKR QKLDIPFIYL TAKTQEHDTI YALTLGADDY VKKPFSPREL VLRINNLLTR
                           ***::.*::   . : **::: ****.*:*.* *.*:*****: : ******** ***::*:* *
TIGR4   SP0083_RR08        LHRGGET--E LISLGNLKMN HSSHEVQIGE EMLDLTVKSF ELLWILASNP ERVFSKTDLY 180 aa
Newman  NWMN0675_SaeR      MKKYHHQPVE QLSFDELTLI NLSKVVTVNG HEVPMRIKEF ELLWYLASRE NEVISKSELL
                           :::  .   *  :*:.:*.:  : *: * :.  . : : :*.* **** ***.  :.*:**::*
TIGR4   SP0083_RR08        EKIWKEDYVD DTNTLNVHIH ALRQELAKYS SDQTPTIKTV WGLGYKIEKP RGQT       234 aa
Newman  NWMN0675_SaeR      EKVWGYDYYE DANTVNVHIH RIREKLEKES -FTTYTITTV WGLGYKFERS R---
                           **:*  ** : *:**:*****  :*::* * *    * **.** ******:*:  *
```
