# Supplementary material for: Pneumococcal Metabolic Adaptation and Colonization Are Regulated by the Two-Component Regulatory System 08
Source: mSphere. 2018 May 16;3(3):e00165-18. doi: 10.1128/mSphere.00165-18 (PMC5956151; doi:10.1128/mSphere.00165-18)
Supplement: TABLE S2 [file sph003182549st2.pdf]

Table S2. Laboratory strains and mutants

***S. pneumoniae* strains**	**characteristics**	**reference**
PN111	D39Δ*cps*	Rennemeier et al., 2007
PN259	TIGR4Δ*cps*	Schulz et al., 2014
PN149	D39*lux*	Jensch et al., 2010
PN315	TIGR4*lux*	Schulz et al., 2014
***S. pneumoniae* mutants**	**characteristics**	**reference**
PN407	D39Δ*cps*Δ*rr08*	This study
PN412	D39Δ*cps*Δ*hk08*	This study
PN308	D39Δ*cps*Δ*tcs08*	This study
PN408	TIGR4Δ*cps*Δ*rr08*	This study
PN652	TIGR4Δ*cps*Δ*hk08*	This study
PN344	TIGR4Δ*cps*Δ*tcs08*	This study
PN409	D39*lux*Δ*rr08*	This study
PN414	D39*lux*Δ*hk08*	This study
PN372	D39*lux*Δ*tcs08*	This study
PN410	TIGR4*lux*Δ*rr08*	This study
PN415	TIGR4*lux*Δ*hk08*	This study
PN708	TIGR4*lux*Δ*tcs08*	This study
***E. coli* strains**	**characteristics**	**reference**
DH5α	Δ(*lac)*U169, *endA1*, *gyrA96*, *hsdR17*, Φ80Δ(*lacZ*)M15 recA1, relA1, supE44, thi-1	Novagen
BL21(DE3)	*E. coli B*, F- dcm *ompT hsdS gal λ* (DE3), T7 polymerase gene under control of the lacUV5 promoter	Stratagene
