# Supplementary material for: Pneumococcal Metabolic Adaptation and Colonization Are Regulated by the Two-Component Regulatory System 08
Source: mSphere. 2018 May 16;3(3):e00165-18. doi: 10.1128/mSphere.00165-18 (PMC5956151; doi:10.1128/mSphere.00165-18)
Supplement: TABLE S3 [file sph003182549st3.pdf]

Table S3. Primers used in this study

Purpose	Primer designation	Sequence (5’-3’)
	**Insertion-deletion mutagenesis**	
Elongation of *hk08* gene	hk08_826	5`-GTGAGGTTCAATACTTATCACC-3`
	hk08_827	5`-CGTTATCCAAACGACGTTCC-3`
Inverse PCR of *hk08* cloned fragment	hk08_824	5`-CCGGGCTGCAGGTTAAGAGGGTTGAAATAATATAT-3`
	hk08_825	5`-CCGGCCTGCAGCCATCAATTGGGTGGGGAAATCA-3`
Elongation of *rr08* gene	rr08_249	5`-GCGCGCGGATCCCATCAGACACAGCAAATGGTTC-3`
	rr08_250	5`-GCGCGCCTGCAGGTGGACATCTCTTCTCTCCTG-3`
Inverse PCR of *rr08* cloned fragment	rr08_984	5`-TCATCGGGTACCCATGGGCTACCAAGACCTGA-3`
	rr08_985	5`-TCACTGCTCGAGTAAGACAGTTTGGGGGTTGG-3`
Elongation of *tcs08* genes	rr08_249	5`-GCGCGCGGATCCCATCAGACACAGCAAATGGTTC-3`
	rr08_250	5`-GCGCGCCTGCAGGTGGACATCTCTTCTCTCCTG-3`
Inverse PCR of *tcs08* cloned fragment	tcs08_252	5`-GCGCGCAAGCTTTTGAACAGCTCTAGCGCTTC-3`
	tcs08_253	5`-GCGCGCAAGCTTTGATTCCTTGGAAGAAAGC-3`
	**Antibiotic cassette preparation**	
Spectinomycin (*aad9*)	spec_117	5'-AAAAAGCTTGAATTCGGATCCATCGATTTTCGTTCGTGAATAC3'
	spec_118	5'-AAAAAGCTTGCTAGCAATTAGAATGAATATTTCCC-3'
Erythromycin (*ermB*)	*ermpstI1_67*	5’-GCGCGCCTGCAGACGGTTCGTGTTCGTGCTG-3’
	Ermpst2_68	5’-GCGCGCCTGCAGCGTAGGCGCTAGGGACCTC-3’
Erythromycin (*ermB*)	*erm*_105	5’-GATGATGATGATCCCGGGTACCAAGCTTGAATTCACGGTTCGTGTTCGTGCTG-3’
	*erm*_106	5’-AGTGAGTGAGTCCCGGGCTCGAGAAGCTTGAATTCGTAGGCGCTAGGGACCTC-3’
	**Real-Time PCR**	
arcA (sp_2148)	*arcA*RT_0611	5’-TGCCGGACTATCTTGAAAGG-3’
	*arcA*RT_1578	5’-GAGAGGTCAATGATTCAGCA-3’
arcD (sp_2152)	RT_*arcD*_F_1676	5’-GGGGCCTTTATAGAAGGTAT-3’
	RT_*arcD*_R_1677	5’-GGCTACATCAATCGCTGCG-3’
psaA (sp_1650)	RT_*psaA*_F_1680	5’-GCTACAAACTCAATCATCGC-3’
	RT_*psaA*_R_1681	5’-GAAAATCAAATTAGCCTCAGAA-3’
pavB (sp_0082)	*pavB*RT_1436	5’-CGGTGTTGCCTCAGTTGTTG-3’
	*pavB*RT_1437	5’-CCCTCAGGTTTCGCTTGAGT-3’
rrgB (sp_0463)	*rrgB*RT_1488	5’-AGCAGAAACGCCTGAAACCA-3’
	*rrgB*RT_1489	5’-CCTGTTTGCGCCTCTGTCC-3’
ribosomal protein S16 (sp_0775)	RT_16S_F	5'-CTACCGTATCAACGTAGCAG-3'
	RT_16S_R	5'-AGTTACTTGGTTTTCAGCAACA-3'
rr08 (sp_0083)	F_qRTPCR_RR08_1702	5'-AGGCAGGTTATCAGGTCTTG-3'
	R_qRTPCR_RR08_1703	5'-TTCACTGGTCTTAGCAGTAATA-3'
hk08 (sp_0084)	F_qRTPCR_HK08_1704	5'-AGGCGAGATTTACTTTTTGCT-3'
	R_qRTPCR_HK08_1705	5'-CTAATTGCTGAAATTCTACAGG-3'
