# Supplementary material for: Pneumococcal Metabolic Adaptation and Colonization Are Regulated by the Two-Component Regulatory System 08
Source: mSphere. 2018 May 16;3(3):e00165-18. doi: 10.1128/mSphere.00165-18 (PMC5956151; doi:10.1128/mSphere.00165-18)
Supplement: TABLE S4 [file sph003182549st4.pdf]

Table S4. Plasmids used in this study

Plasmids	Features	Source
	**Commercial plasmids**	
pGEM-T Easy	Cloning Vector (3016bp), Amp^r	Promega
pSP72	Cloning Vector (2462bp), Amp^r	Promega
	**Mutagenesis plasmids**	
p927	pGEM-T Easy variant with *sp_0084* construct for mutagenesis	This study
p928	pGEM-T Easy variant with *sp_0083* construct for mutagenesis	This study
p896	pGEM-T Easy variant with *sp_0083+sp_0084* construct for mutagenesis	This study
