# Supplementary material for: Pneumococcal Metabolic Adaptation and Colonization Are Regulated by the Two-Component Regulatory System 08
Source: mSphere. 2018 May 16;3(3):e00165-18. doi: 10.1128/mSphere.00165-18 (PMC5956151; doi:10.1128/mSphere.00165-18)
Supplement: TABLE S5 [file sph003182549st5.pdf]

Table S5. *In silico* search for RR08 binding motifs

TIGR4 Locus_Tag	*TIGR4 new Locus_Tag*	binding motif search (5'-3')	Genomic position (bp)
sp_0082	sp_rs00425	tataa-N6-tttaa-N7-tttaa-N16-tttaa	86,193
sp_0083	sp_rs00430	aaatt-N39-aattt-N32-aattt	88,916
sp_0084	sp_rs00435	ND	NA
sp_0085	sp_rs00440	ND	NA
sp_0090	sp_rs00460	ttaaa-N40-ttaaa-N159-tttaaa-N41-tttaa	92,642
sp_0091	sp_rs00465	ND	NA
sp_0092	sp_rs00475	ND	NA
sp_0101	sp_rs00510	aaatt-N67-tataa-N56-aaatt-N6-tataa	102,070
sp_0111	sp_rs00565	ttaaa-N8-ttaaa-N48-aaatt-N15-aaatt-N65-tttaa	112,758
sp_0112	sp_rs00570	atatt-N36-atatt-N28-tttaa-N8-aaatt-N2-aattt	113,913
sp_0144	sp_rs00740	tttaa-N104-gttaa	141,586
sp_0148	sp_rs00765	tttaa-N39-tataa-N21-gttaa	145,404
sp_0198	sp_rs00960	aaatt-N207-aaatt-N-48-aaatt-N5-aaatt-N13-aaatt	184,314
sp_0247	sp_rs01200	gttaa-N16-gttaa	216,720
sp_0248	sp_rs01205	tataa-N76-tttaa	217,791
sp_0249	sp_rs01210	tttaa-N6-aaatt-N55-ttaac	218,197
sp_0250	sp_rs01215	aaatt-N51-aaatt-N74-aaatt	218,354
sp_0251	sp_rs01220	gttaa-N29-gttaa-N10-gttaa	219,933
sp_0278	sp_rs01355	atatt-N37-caatt-N17-caatt	255,677
sp_0317	sp_rs01540	ND	NA
sp_0318	sp_rs01545	tttaa-N41-tttaa	293,621
sp_0319	sp_rs01550	ND	NA
sp_0320	sp_rs01555	gttaa-N8-tataa-N51-tataa-N48-ttaaa-N3-gttaa-N26-tttaa-N43-tataa	294,998
sp_0321	sp_rs01560	ttaaa-N3-gttaa-N26-tttaa-N43-tataa	295,120
sp_0322	sp_rs01565	tttaa-N106-gttaa-N44-gttaa	295,417
sp_0323	sp_rs01570	tttaa-N91-ttaaa-N22-tttaa-N64-tttaa	296,377
sp_0324	sp_rs01575	tttaa-N149-tttaa-N59-tttaa	297,163
sp_0325	sp_rs01580	tttaa-N18-aattt	297,811
sp_0369	sp_rs01800	gttaa-N10-ttaac-N115-ttaaa-N46-tttaa-N40-tataa-N155-ttaac-N82-gttaa	346,311
sp_0394	sp_rs01955	tataa-N2-tttaa-N63-tttaa-N5-tttaa-N16-tttaa	372,180
sp_0395	sp_rs01960	gttaa-N184-ttaac-N74-ttaac-N118-aaatt-N15-tttaa	373,521
sp_0396	sp_rs01965	tttaa-N37-ttata-N56-tttaa	375,922
sp_0397	sp_rs01970	aaatt-N7-aaatt-N31-ttaaa	376,387
sp_0415	sp_rs02050	tttaa-N92-tttaa-N45-tttaa-N40-ttata-N141-tttaa-N89-tttaa-N16-tataa-N6-tataa	393,858
sp_0416	sp_rs02055	tttaa-N81-ttaaa-N63-tttaa-N106-aaatt-N35-aattt-N3-tataa	394,847
sp_0417	sp_rs02060	tttaa-N8-tttaa-N43-tttaa-N121-aattt-N128-aaatt-N22-aattt-N34-aattt	395,249
sp_0418	sp_rs02065	tataa-N13-tttaa	396,656

sp_0419	sp_rs02070	ND	NA
sp_0420	sp_rs02075	gttaa-N177-ttaac-N183-tttaa-N60-ttaaa-N42-tttaa	397,298
sp_0421	sp_rs02080	ND	NA
sp_0422	sp_rs02085	ND	NA
sp_0423	sp_rs02090	ND	NA
sp_0424	sp_rs02095	ND	NA
sp_0425	sp_rs02100	tttaa-N133-gttaa	401,557
sp_0426	sp_rs02105	gttaa-N37-gttaa-N64-tttaa-N87-ttata-N239-tttaa-N3-tttaa	402,789
sp_0427	sp_rs02110	ND	NA
sp_0461	sp_rs02275	tttaa-N4-tataa-N38-aattt-N38-tttaa-N79-tataa-N41-tttaa-N15-gttaa-N62-tataa-N7-ttata-N29-ttaac-N83-ttaac-N18-ttaac	437,864
sp_0462	sp_rs02280	tttaa-N4-tataa-N38-aattt-N38-tttaa-N79-tataa-N41-tttaa-N15-gttaa-N62-tataa-N7-ttata-N29-ttaac-N83-ttaac-N18-ttaac	437,864
sp_0463	sp_rs02285	tttaa-N21-tttaa-N127-tttaa	441,046
sp_0464	sp_rs02290	ND	NA
sp_0465	#N/A	ND	NA
sp_0466	sp_rs02295	ND	NA
sp_0467	sp_rs02300	ND	NA
sp_0468	sp_rs02305	ND	NA
SP_0476	sp_rs02350	tttaa-N5-tttaa-N43-tttaa	455,401
SP_0477	sp_rs02355	ND	NA
sp_0478	sp_rs02360	tttaa-N5-tttaa	457,294
sp_0479	sp_rs02365	tttaa-N15-tttaa	459,076
sp_0480	sp_rs02370	ND	NA
sp_0507	sp_rs02490	ND	NA
sp_0508	sp_rs02495	ttaac-N41-ttaac-N18-tataa-N37-ttata	488,567
sp_0516	sp_rs02525	ND	NA
sp_0517	sp_rs02530	aattt-N123-aattt-N-106-ttaac	497,222
sp_0518	#N/A	ND	NA
sp_0519	sp_rs02540	tataa-N12-ttata-N5-tttaa-N12-gttaa-N144-tataa	499,680
sp_0605	sp_rs02975	gttaa-N54-tttaa	571,361
sp_0607	sp_rs02985	ND	NA
sp_0608	sp_rs02990	tttaa-N23-gttaa	574,859
sp_0609	sp_rs02995	ND	NA
sp_0611	sp_rs03005	tttaa-N41-tataa-N46-aaatt-N101-tataa-N5-tataa-N37-tataa-N	577,154
sp_0620	sp_rs03045	ND	NA
sp_0641	sp_rs03145	tttaa-N18-ttaac-N5-gttaa-N10-ttaaa-N4-tttaa-N31-ttatatataa-N42-tataa-N14-gttaa-N23-tataa-N12-ttaaa	603,681
sp_0646	sp_rs03160	ND	NA
sp_0647	sp_rs03165	ttaaa-N8-attaa-N14-gttaa	613,501
sp_0668	sp_rs03280	ttaac-N13-aaatt-N42-ttaac	643,880
sp_0709	sp_rs03470	ND	NA
sp_0710	sp_rs03475	ND	NA
sp_0711	sp_rs03480	ttaaa-N79-gttaa	675,224

sp_0743	sp_rs03640	gttaa-N21-gttaa-N63-aaatt-N15-ttata-N30-ttata	704,730
sp_0783	sp_rs03825	gttaa-N25-ttata-N28-tttaa-N79-aattt-N16-ttata-N	736,712
sp_0798	sp_rs03905	tttaa-N4-aattt-N3-tataa-N4-tttaa	751,915
sp_0799	sp_rs03910	ttaaa-N36-tttaa-N-31-gttaa-N16-gttaa	752,283
sp_0899	sp_rs04440	ttaaa-N6-ttaag-N43-ttaaa-N31-gttaa-N6-tttaa	854,511
sp_0912	sp_rs04505	ttata-N19-gttaa-N94-tttaa-N36-ttata-N28-ttaaa-N127-aaatt	864,298
sp_0913	sp_rs04510	ttaaa-N54-tttaa-N19-gttaa-N39-tataa-N14-aaatt-N26-gttaa-	864,932
sp_0999	sp_rs04955	ND	NA
sp_1032	sp_rs05120	tttaa-N43-tttaa-N46-tttaa-N132-gttaa-N18-tttaa-N34-ttata-N3-ttaaa-N59-tttaa	973,021
sp_1033	sp_rs05125	tttaa-N29-ttaaa-N32-aaatt-N112-ttaaa	974,106
sp_1034	sp_rs05130	tttaa-N16-tttaa-N26-aaatt-N108-tttaa-N41-gttaa-N1-gttaa-N86-ttata-N22-ttata-N37-ttata-N16-ttata-N5-tataa	974,902
sp_1035	sp_rs05135	tttaa-N16-tttaa-N65-aattt	976,158
sp_1185	sp_rs05830	ttaaa-N15-tttaa	1,124,503
sp_1186	sp_rs05835	ttata-N4-tttaa-N57-ttata-N75-tttaa	1,124,805
sp_1197	sp_rs05885	tttaa-N57-aattt-42-ttaac	1,131,638
sp_1198	sp_rs5890	ttaac-Ntataa-N1-tataa-N17-gttaa_N29-ttata-N12-ttata-N5-ttata	1,132,148
sp_1199	#N/A	ND	NA
sp_1282	sp_rs06285	aattt-N18-aattt-N2-ttaaa-N2-tataa-N0-tataa-N123-gttaa-N225-ttaaa-N2-tttaa	1,216,096
sp_1315	sp_rs06455	ttaaa-N47-tttaa-N67-ttata	1,239,170
sp_1316	sp_rs06460	tataa-N7-ttata-N49-tataa	1,240,544
sp_1317	sp_rs06465	tttaa-N7-tttaa	1,242,443
sp_1318	sp_rs06470	ND	NA
sp_1319	sp_rs06475	aattt-N14-aattt-N11-ttaac	1,243,725
sp_1320	sp_rs06480	gttaa-N11-ttaaa	1,244,620
sp_1321	sp_rs06485	ttaaa-N4-tttaa-N34-tttaa	1,245,601
sp_1322	sp_rs06490	ND	NA
sp_1326	sp_rs06510	tttaa-N27-tttaa-N18-tttaa	1,251,613
sp_1341	sp_rs06580	tttaa-N38-aattt-N23-ttata-N22-tttaa-N62-aattt-N15-aaatt	1,263,996
sp_1342	sp_rs11490	tataa-N23-tttaa-N11-ttaaa-N21-tttaa	1,264,708
sp_1346	sp_rs06600	tttaa-N19-gttaa-N19-ttaaa	1,271,721
sp_1434	sp_rs07035	attaa-N22-ttata-N38-attaa-N6-ttata-N12-attaa	1,352,482
sp_1435	sp_rs07040	ttaaa-N14-tttaa-N100-tttaa	1,353,934
sp_1458	sp_rs07165	ND	NA
sp_1459	sp_rs07170	tttaa-N45-ttata	1,375,544
sp_1460	sp_rs07175	ND	NA
sp_1461	sp_rs07180	aaatt-N64-ttata	1,377,196
sp_1462	sp_rs07185	ND	NA
sp_1492	sp_rs07345	ND	NA
sp_1527	sp_rs07525	tttaa-N12-ttata	1,439,510
sp_1528	#N/A	ND	NA
sp_1647	sp_rs08130	ttaac-N31-tttaa-N17-ttata-N61-tataa-N6-aattt-N42-tttaa-N12-ttaac-N16-tttaa	1,547,610

sp_1648	sp_rs08135	tttaa-N12-ttaac-N16-tttaa	1,547,791
sp_1649	sp_rs08140	ND	NA
sp_1650	sp_rs08145	ND	NA
sp_1823	sp_rs09045	gttaa-N6-gttaa-N2-tttaa	1,734,445
sp_1824	sp_rs09050	tttaa-20-ttaac-N2-tttaa	1,736,639
sp_1825	sp_rs09055	tttaa-N16-tttaa	1,736,936
sp_1826	sp_rs09060	aaatt-N3-gttaa	1,738,258
sp_1830	sp_rs09080	ttaac-N16-ttaac-N25-tttaa-N75-ttaaa-N11-ttaac-N55-ttaaa	1,742,145
sp_1856	sp_rs09210	tttaa-N66-ttata-N57-ttaac-N17-ttata-N18-aattt-N8-tttaa	1,764,323
sp_1869	sp_rs09270	ttaaa-N8-aattt-N29-ttata-N62-tttaa	1,774,413
sp_1870	sp_rs09275	ND	NA
sp_1871	sp_rs09280	ttata-N52-ttata-N27-ttaac	1,776,205
sp_1872	sp_rs09285	tttaa-N25-ttaaa	1,777,244
sp_1884	sp_rs09350	ttaaa-N16-tttaa-N30-tttaa	1,788,798
sp_1885	sp_rs09355	ttaaa-N16-tttaa-N30-tttaa	1,788,798
sp_1894	sp_rs09405	tttaa-N123-tttaa	1,800,976
sp_1895	sp_rs09415	ttaaa-N47-taatt-N48-tttaa-N9-tataa	1,802,243
sp_1896	sp_rs09420	gttaa-N43-ttaaa-N16-ttaat	1,803,475
sp_1897	sp_rs09425	gttaa-N52-ttata-N22-tttaa-N1-tttaa	1,804,532
sp_2006	sp_rs10155	ND	NA
sp_2022	sp_rs10230	tttaa-N22-gttaa	1,927,192
sp_2023	sp_rs10235	ttaac-N80-tttaa-N29-ttaat-N112-ttata-N46-tttaa-N42-ttata-N27-ttaaa-N94-gttaa-N29-gttaa	1,928,668
sp_2024	sp_rs10240	gttaa-N29-gttaa-N12-aattt-N38-gttaa	1,929,132
sp_2106	sp_rs10735	gttaa-N117-tttaa-N16-ttaat	2,018,567
sp_2107	sp_rs10740	gttaa-N46-ttata-N34-tttaa-N18-tttaa-N29-ttaaa	2,020,215
sp_2108	sp_rs10745	ttaaa-N37-ttaaa-N27-ttaaa-N5-tttaa	2,020,544
sp_2129	sp_rs10865	ttaaa-N65-tataa-N27-ttaat-N30-tttaa	2,041,877
sp_2130	sp_rs10870	tttaa-N18-tttaa-N7-tttaa-N24-ttaaa-N22-ttaaa-N6-gttaa-N13-tttaa	2,042,221
sp_2136	sp_rs10900	gttaa-N23-tttaa-N36-ttata-N16-ttata-N45-ttata-N34-tttaa-N2-tttaa-N11-ttaac-N4-tttaa-N7-tttaa-N5-gttaa-N8-ttaaa_N4-gttaa-N8-ttaac	2,048,492
sp_2148	sp_rs10955	tttaa-N7-ttata-N6-ttata-N43-tataa-N29-ttata-N15-ttata-N26-ttata-N43-tttaa	2,061,922
sp_2150	sp_rs10960	ND	NA
sp_2151	sp_rs10965	tataa-N16-ttaaa	2,064,541
sp_2152	sp_rs10970	tttaa-N45-tttaa	2,065,663
sp_2153	sp_rs10975	tataa-N32-tataa-N9-ttaaa-N18-ttaaa	2,067,274
sp_2184	sp_rs11155	ttaaa-N48-ttaaa-N58-tttaa	2,102,709
sp_2185	sp_rs11160	ND	NA
sp_2186	sp_rs11165	tttaa-N2-tttaa-N44-ttaac-N7-tttaa-N51-tttaa	2,106,284
sp_2235	sp_rs11425	ttaaa-N20-ttaaa-N85-tttaa	2,156,404
sp_2236	sp_rs11430	tataa-N29-ttaaa-N19-ttaaa	2,157,638
sp_2237	sp_rs11435	tttaa-N43-tttaa-N63-ttata-N66-tttaa	2,157,819
